# Prognostic impact of elective tracheotomy in resected oral cavity squamous cell carcinoma: A nationwide cohort study

**DOI:** 10.1002/cam4.7213

**Published:** 2024-06-18

**Authors:** Ku‐Hao Fang, Chung‐Jan Kang, Li‐Yu Lee, Shu‐Hang Ng, Chien‐Yu Lin, Wen‐Cheng Chen, Jin‐Ching Lin, Yao‐Te Tsai, Shu‐Ru Lee, Chih‐Yen Chien, Chun‐Hung Hua, Cheng Ping Wang, Tsung‐Ming Chen, Shyuang‐Der Terng, Chi‐Ying Tsai, Hung‐Ming Wang, Chia‐Hsun Hsieh, Kang‐Hsing Fan, Chih‐Hua Yeh, Chih‐Hung Lin, Chung‐Kan Tsao, Nai‐Ming Cheng, Tuan‐Jen Fang, Shiang‐Fu Huang, Li‐Ang Lee, Yu‐Chien Wang, Wan‐Ni Lin, Li‐Jen Hsin, Tzu‐Chen Yen, Yu‐Wen Wen, Chun‐Ta Liao

**Affiliations:** ^1^ Department of Otorhinolaryngology, Head and Neck Surgery Chang Gung Memorial Hospital and Chang Gung University Taoyuan Taiwan, ROC; ^2^ Department of Pathology Chang Gung Memorial Hospital and Chang Gung University Taoyuan Taiwan, ROC; ^3^ Department of Diagnostic Radiology Chang Gung Memorial Hospital and Chang Gung University Taoyuan Taiwan, ROC; ^4^ Department of Radiation Oncology Chang Gung Memorial Hospital and Chang Gung University Taoyuan Taiwan, ROC; ^5^ Department of Radiation Oncology Chang Gung Memorial Hospital and Chiayi and Chang Gung University Taoyuan Taiwan, ROC; ^6^ Department of Radiation Oncology Changhua Christian Hospital Changhua Taiwan, ROC; ^7^ Department of Otorhinolaryngology‐Head and Neck Surgery Chang Gung Memorial Hospital Chiayi Taiwan, ROC; ^8^ Research Service Center for Health Information Chang Gung University Taoyuan Taiwan, ROC; ^9^ Department of Otolaryngology, Chang Gung Memorial Hospital Kaohsiung Medical Center Chang Gung University College of Medicine Taoyuan Taiwan, ROC; ^10^ Department of Otorhinolaryngology China Medical University Hospital Taichung Taiwan, ROC; ^11^ Department of Otolaryngology National Taiwan University Hospital and College of Medicine Taipei Taiwan, ROC; ^12^ Department of Otolaryngology, Shuang Ho Hospital Taipei Medical University New Taipei City Taiwan, ROC; ^13^ Department of Head and Neck Surgery Koo Foundation Sun Yat‐Sen Cancer Center Taipei Taiwan, ROC; ^14^ Department of Oral and Maxillofacial Surgery, Chang Gung Memorial Hospital Chang Gung University Taoyuan Taiwan, ROC; ^15^ Department of Medical Oncology, Chang Gung Memorial Hospital and Chang Gung University Taoyuan Taiwan, ROC; ^16^ Department of Radiation Oncology New Taipei Municipal TuCheng Hospital New Taipei Taiwan, ROC; ^17^ Department of Plastic and Reconstructive Surgery Chang Gung Memorial Hospital and Chang Gung University Taoyuan Taiwan, ROC; ^18^ Department of Nuclear Medicine and Molecular Imaging Center Chang Gung Memorial Hospital and Chang Gung University Taoyuan Taiwan, ROC; ^19^ Department of Biomedical Sciences, College of Medicine Chang Gung University Taoyuan Taiwan, ROC; ^20^ Division of Thoracic Surgery Chang Gung Memorial Hospital Taoyuan Taiwan, ROC

**Keywords:** cancer registry, clinical outcomes, oral cavity squamous cell carcinoma, tracheotomy

## Abstract

**Background:**

Elective tracheotomy is commonly performed in resected oral squamous cell carcinoma (OCSCC) to maintain airway patency. However, the indications for this procedure vary among surgeons. This nationwide study evaluated the impact of tracheotomy on both the duration of in‐hospital stay and long‐term survival outcomes in patients with OCSCC.

**Methods:**

A total of 18,416 patients with OCSCC were included in the analysis, comprising 7981 patients who underwent elective tracheotomy and 10,435 who did not. The primary outcomes assessed were 5‐year disease‐specific survival (DSS) and overall survival (OS). To minimize potential confounding factors, a propensity score (PS)‐matched analysis was performed on 4301 patients from each group. The duration of hospital stay was not included as a variable in the PS‐matched analysis.

**Results:**

Prior to PS matching, patients with tracheotomy had significantly lower 5‐year DSS and OS rates compared to those without (71% vs. 82%, *p* < 0.0001; 62% vs. 75%, *p* < 0.0001, respectively). Multivariable analysis identified tracheotomy as an independent adverse prognostic factor for 5‐year DSS (hazard ratio = 1.10 [1.03–1.18], *p* = 0.0063) and OS (hazard ratio = 1.10 [1.04–1.17], *p* = 0.0015). In the PS‐matched cohort, the 5‐year DSS was 75% for patients with tracheotomy and 76% for those without (*p* = 0.1488). Five‐year OS rates were 66% and 67%, respectively (*p* = 0.0808). Prior to PS matching, patients with tracheotomy had a significantly longer mean hospital stay compared to those without (23.37 ± 10.56 days vs. 14.19 ± 8.34 days; *p* < 0.0001). Following PS matching, the difference in hospital stay duration between the two groups remained significant (22.34 ± 10.25 days vs. 17.59 ± 9.54 days; *p* < 0.0001).

**Conclusions:**

While elective tracheotomy in resected OCSCC patients may not significantly affect survival, it could be associated with prolonged hospital stays.

## Graphical abstract

Our study indicates that, following propensity score matching, patients with oral cavity squamous cell carcinoma (OCSCC) who underwent tracheotomy exhibit similar 5‐year disease‐specific and overall survival rates compared to those who did not. While elective tracheotomy in resected OCSCC patients may not significantly affect survival, it could be associated with prolonged hospital stays.

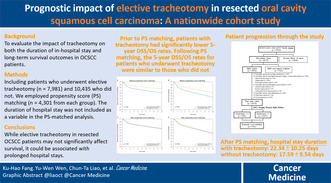

## 1 INTRODUCTION

The primary treatment strategy for oral squamous cell carcinoma (OCSCC) involves extensive tumor resection, which may be paired with neck dissection.^1^, ^2^ While it is common practice to perform an elective tracheotomy during the tumor resection procedure,^3^, ^4^, ^5^, ^6^, ^7^, ^8^, ^9^, ^10^, ^11^, ^12^, ^13^, ^14^ the decision to employ this approach is not uniform among surgeons. Consequently, the reported incidence of elective tracheotomy in patients who have undergone OCSCC excision varies significantly in the literature, with a range of 14%–60%.^5^, ^6^, ^12^, ^13^ In scenarios involving extensive resection of OCSCC, certain practitioners recommend the execution of an elective tracheotomy in all cases where free flap reconstruction is required.^9^ Furthermore, some researchers have proposed the implementation of a tracheotomy scoring system to provide a logical basis for this procedure during OCSCC surgery. This strategy has led to tracheotomy rates that fluctuate between 14%^12^ and 34%.^13^ Although elective tracheotomy is associated with certain complications^7^, ^8^, ^9^, ^11^ and has been reported to influence survival rates,^10^ some researchers have observed that surgeries for OCSCC, which often necessitate tracheotomy, do not typically lead to severe complications or significantly affect the overall outcomes.^9^, ^11^

Anecdotal evidence from our clinical practice suggests that tracheostomy may not have a substantial influence on the survival outcomes of patients with OCSCC. However, we have noted variations in the indications and decision‐making processes among our surgeons when considering tracheostomy. At present, there are no large‐scale studies that specifically address the outcomes of tracheotomy in patients with OCSCC, as staged by the American Joint Committee on Cancer (AJCC) Staging Manual, Eighth Edition.^14^ To delve deeper into this issue, we carried out a large‐scale, nationwide study in Taiwan to assess the effects of tracheotomy on both in‐hospital stay length and survival rates for OCSCC patients. We hypothesized that there would be a significant difference in the length of hospital stay between OCSCC patients who had undergone tracheotomy and those who had not. However, we expected the difference in survival rates between these two groups to be minimal.

## 2 METHODS

### 2.1 Data sources

The current study follows the Reporting Recommendations for Tumor Marker Prognostic Studies (REMARK)^15^, ^16^ guidelines and was approved by the Chang Gung Memorial Hospital Ethics Committee (reference number: 201801398B0A3). A waiver of informed consent was granted. We sourced patient data from the extensive “long‐form” of the Taiwanese Cancer Registry Database (TCRD), covering over 99% of Taiwanese patients diagnosed with OCSCC. However, the TCRD does not provide information on salvage therapy for patients who experience disease relapse. We obtained survival outcome data from the Taiwanese National Health Insurance Research Dataset (TNHIRD). While the TNHIRD includes data on whether patients underwent reconstruction with free flaps, it does not provide granular details on procedures such as mandibulotomy, mandibulectomy, or maxillectomy.

### 2.2 Patient selection

**FIGURE 1:**
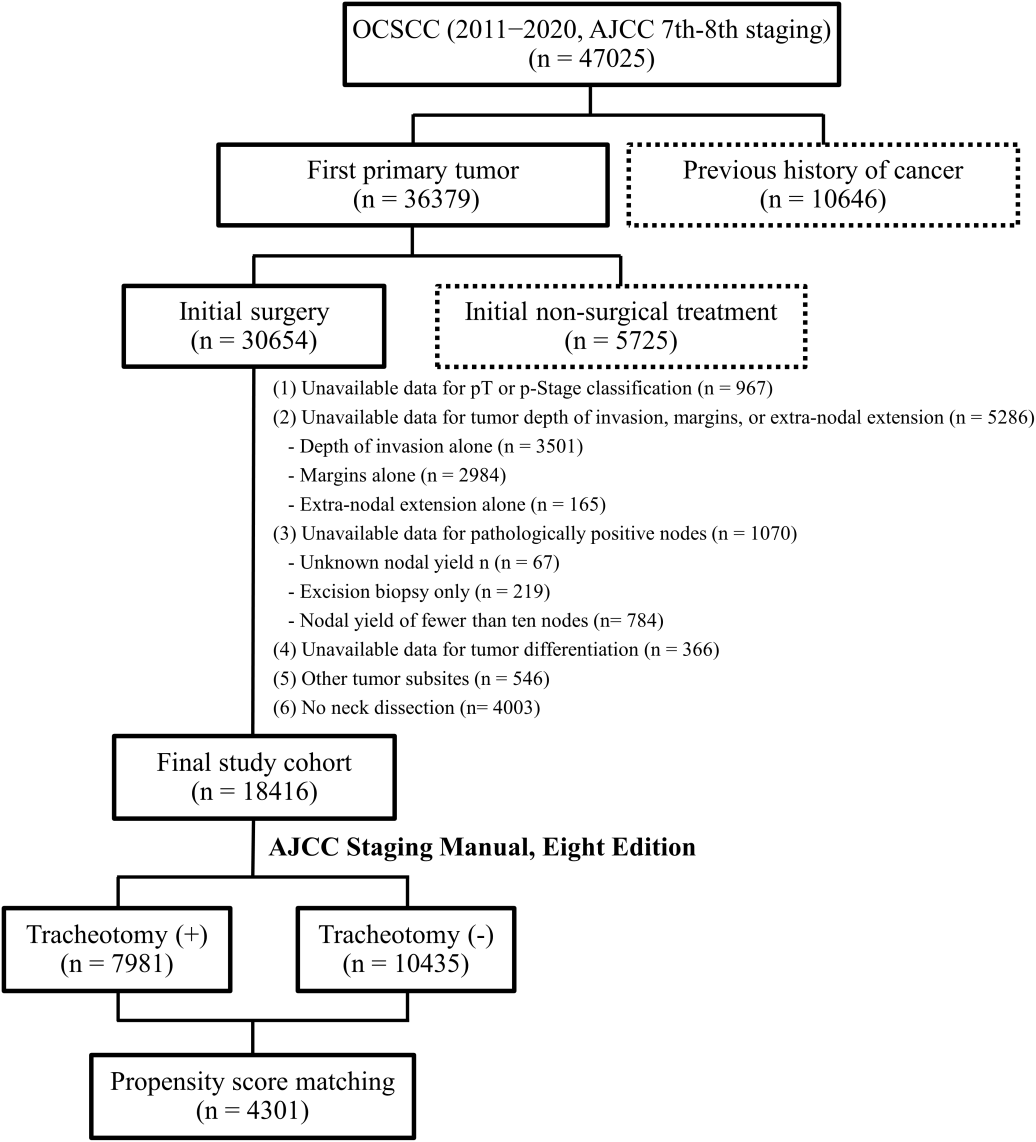
Patient progression through the study.

Between 2011 and 2020, a total of 47,025 patients diagnosed with OCSCC were considered for inclusion in this study. Case selection was based on the International Classification of Diseases for Oncology, Third Edition (ICD‐O‐3) codes. The study flow chart (Figure 1) provides details on case inclusion and exclusion. Patients were excluded if their medical records indicated: (1) a prior diagnosis of cancer (*n* = 10,646), (2) initial non‐surgical treatment (*n* = 5725), (3) unknown pathological stage (*n* = 967), (4) unavailable data on tumor depth, surgical margins, or extra‐nodal extension (ENE) (*n* = 5286), (5) unavailable information on pathological lymph node metastases or a nodal yield of fewer than 10 nodes (*n* = 1070), (6) no information on tumor differentiation (*n* = 366), (7) tumor site other than the oral cavity (*n* = 546), and (8) no neck dissection performed (*n* = 4003). The final study cohort consisted of 18,416 patients, all of Taiwanese descent. Initial staging was based on the AJCC Staging Manual, Seventh Edition (2010). However, an updated classification was later obtained in line with the AJCC Staging Manual, Eighth Edition (2018), taking into account the depth of invasion (DOI) and ENE.^17^ Among the 18,416 study patients with OCSCC included in the study, 43.3% (*n* = 7981) underwent tracheotomy, whereas 56.7% (*n* = 10,435) did not. The follow‐up period was calculated from the day of surgery until either the patient's death or the end of the study in December 2021.

### 2.3 Data collection

The study variables were acquired from the 2020 version of the TCRD and the 2021 version of the TNHIRD. The final data analysis was carried out in October 2023. The TCRD complies with the guidelines outlined in the Standards for Oncology Registry Entry (STORE) manual.^18^ Data pertaining to the morbidity and mortality associated with OCSCC were gathered from the TNHIRD. This information was then utilized to calculate disease‐specific survival (DSS) and overall survival (OS), respectively.

### 2.4 Statistical analysis

To visualize survival estimates, we generated Kaplan–Meier plots and used the log‐rank test for statistical comparison. To delve into the relationships between the variables under study and survival outcomes, we conducted both univariable and multivariable Cox proportional hazards regression analyses. We adopted a stepwise selection approach, incorporating all variables from the univariable analysis into the multivariable model. The results are presented as hazard ratios (HRs) with their corresponding 95% confidence intervals (CIs). All statistical tests were two‐sided and performed at a 5% significance level.

## 3 RESULTS

### 3.1 Patient characteristics

Table 1 presents the general characteristics of OCSCC patients who underwent tracheotomy and those who did not. The tracheotomy group exhibited a significantly higher prevalence of several variables compared to the non‐tracheotomy group (all *p* values <0.0001). These factors included: (1) tumors located at the gum, mouth floor, retromolar subsites, (2) male sex, (3) pT3–4 disease, (4) pN2–3 disease, (5) p‐Stage IV, (6) poorly differentiated tumor, (7) clear margins (≥5 mm), (8) deeper DOI, (9) reconstruction with free flap reconstruction, (10) treatment involving surgery and adjuvant therapy, (11) higher weighted Charlson comorbidity index (CCI),^19^ and (12) prolonged hospital stays.

**TABLE 1:** General characteristics of oral cavity squamous cell carcinoma patients with and without tracheotomy before and after propensity score matching.

Characteristic (*n*, %)	Before propensity score matching (*n* = 18,416)				After propensity score matching (*n* = 8602)			
	Non‐tracheotomy (*n* = 10,435)	Tracheotomy (*n* = 7981)	SMD (%)	*p*	Non‐tracheotomy (*n* = 4301)	Tracheotomy (*n* = 4301)	SMD (%)	*p*
Tumor subsite								
Lip (406, 2.2)	303 (2.9)	103 (1.3)	11.28	<0.0001	104 (2.4)	95 (2.2)	1.39	0.1332
Tongue (7232, 39.3)	4511 (43.2)	2721 (34.1)	18.84		1251 (29.1)	1338 (31.1)	−4.41	
Gum (2714, 14.7)	1112 (10.7)	1602 (20.1)	−26.34		719 (16.7)	744 (17.3)	−1.55	
Mouth floor (731, 4.0)	309 (3.0)	422 (5.3)	−11.72		162 (3.8)	155 (3.6)	0.86	
Hard palate (226, 1.2)	86 (0.8)	140 (1.8)	−8.25		47 (1.1)	55 (1.3)	−1.72	
Buccal (6295, 34.2)	3809 (36.5)	2486 (31.1)	11.33		1813 (42.2)	1696 (39.4)	5.54	
Retromolar (812, 4.4)	305 (2.9)	507 (6.3)	−16.36		205 (4.7)	218 (5.1)	−1.40	
Sex								
Men (16,761, 91.0)	9308 (89.2)	7453 (93.4)	−14.88	<0.0001	4020 (93.5)	3977 (92.5)	3.91	0.0655
Women (1655, 9.0)	1127 (10.8)	528 (6.6)	14.88		281 (6.5)	324 (7.5)	−3.91	
Age (years)	55.00 ± 11.08	55.20 ± 10.57	−1.85	0.2172	54.99 ± 10.60	55.04 ± 10.75	−0.47	0.8115
< 65 (14,947, 81.2)	8432 (80.8)	6515 (81.6)	−2.12	0.1552	3519 (81.8)	3521 (81.9)	−0.12	0.9548
≥ 65 (3469, 18.8)	2003 (19.2)	1466 (18.4)	2.12		782 (18.2)	780 (18.1)	0.12	
Pathologic T status								
T1 (3693, 20.1)	3179 (30.5)	514 (6.4)	65.13	<0.0001	511 (11.9)	501 (11.6)	0.72	0.832
T2 (5854, 31.8)	4129 (39.6)	1725 (21.6)	39.73		1431 (33.3)	1462 (34.0)	−1.48	
T3 (2220, 12.1)	1103 (10.6)	1117 (14.0)	−10.45		697 (16.2)	675 (15.7)	1.40	
T4 (6649, 36.0)	2024 (19.4)	4625 (58.0)	−86.21		1662 (38.6)	1663 (38.7)	−0.05	
Pathologic N status								
pN0 (12,330, 67.0)	7586 (72.7)	4744 (59.4)	28.28	<0.0001	2791 (64.9)	2790 (64.9)	0.05	0.9426
pN1 (1782, 9.7)	1034 (9.9)	748 (9.4)	1.82		440 (10.2)	425 (9.9)	1.16	
pN2 (1996, 10.8)	988 (9.5)	1008 (12.6)	−10.1		514 (12.0)	521 (12.1)	−0.50	
pN3 (2308, 12.5)	827 (7.9)	1481 (18.6)	−31.76		556 (12.9)	565 (13.1)	−0.62	
Pathologic stage								
I (3226, 17.5)	2789 (26.7)	437 (5.5)	60.4	<0.0001	436 (10.1)	428 (10.0)	0.62	0.9557
II (4213, 22.9)	3009 (28.8)	1204 (15.1)	33.68		1027 (23.9)	1037 (24.1)	−0.54	
III (2421, 13.1)	1473 (14.1)	948 (11.9)	6.66		679 (15.8)	666 (15.5)	0.83	
IV (8556, 46.5)	3164 (30.4)	5392 (67.5)	−80.27		2159 (50.2)	2170 (50.4)	−0.51	
Tumor differentiation								
Well differentiated (4956, 26.9)	2906 (27.8)	2050 (25.7)	4.89	<0.0001	1091 (25.4)	1096 (25.5)	−0.27	0.8499
Moderately differentiated (11,838, 64.3)	6687 (64.1)	5151 (64.5)	−0.96		2818 (65.5)	2799 (65.1)	0.93	
Poorly differentiated (1622, 8.8)	842 (8.1)	780 (9.8)	−5.98		392 (9.1)	406 (9.4)	−1.12	
Margin status (mm)								
Positive (1013, 5.5)	493 (4.7)	520 (6.5)	−7.78	<0.0001	261 (6.1)	251 (5.8)	0.98	0.7832
< 5 (8375, 45.5)	5038 (48.3)	3337 (41.8)	13.03		1857 (43.2)	1839 (42.8)	0.85	
≥ 5 (9028, 49.0)	4904 (47.0)	4124 (51.7)	−9.37		2183 (50.7)	2211 (51.4)	−1.30	
Depth of invasion (mm)								
Mean ± standard deviation	7.89 ± 6.95	14.33 ± 10.10	−74.29	<0.0001	10.98 ± 8.62	11.02 ± 8.16	−0.48	0.7876
Free flap reconstruction								
No (7475, 40.6)	6461 (61.9)	1014 (12.7)	118.19	<0.0001	961 (22.3)	1005 (23.4)	−2.44	0.082
Yes (10,941, 59.4)	3974 (38.1)	6967 (87.3)	−118.19		3340 (77.7)	3296 (76.6)	2.44	
Treatment modality								
S alone (8878, 48.2)	6326 (60.6)	2552 (32.0)	59.98	<0.0001	1882 (43.8)	1859 (43.2)	1.08	0.5795
S plus adjuvant therapy (9538, 51.8)	4109 (39.4)	5429 (68.0)	−59.98		2419 (56.2)	2442 (56.8)	−1.08	
	
Mean ± standard deviation	0.75 ± 1.10	0.78 ± 1.13	−2.69	0.0255	0.75 ± 1.10	0.79 ± 1.15	−3.55	0.1085
0 (10,071, 54.7)	5769 (55.3)	4302 (53.9)	2.78	0.0619	2346 (54.5)	2331 (54.2)	0.70	0.7373
≥1 (8345, 45.3)	4666 (44.7)	3679 (46.1)	−2.78		1955 (45.5)	1970 (45.8)	−0.70	
Length of hospital stay (days) Median, Mean ± standard deviation	12.0, 14.19 ± 8.34	21.0, 23.37 ± 10.56	−96.48	<0.0001	17.59 ± 9.54	22.34 ± 10.25	−60.43	<0.0001

Abbreviations: SMD, standardized mean difference; S, surgery.

### 3.2 Tracheotomy rates across surgeons

**FIGURE 2:**
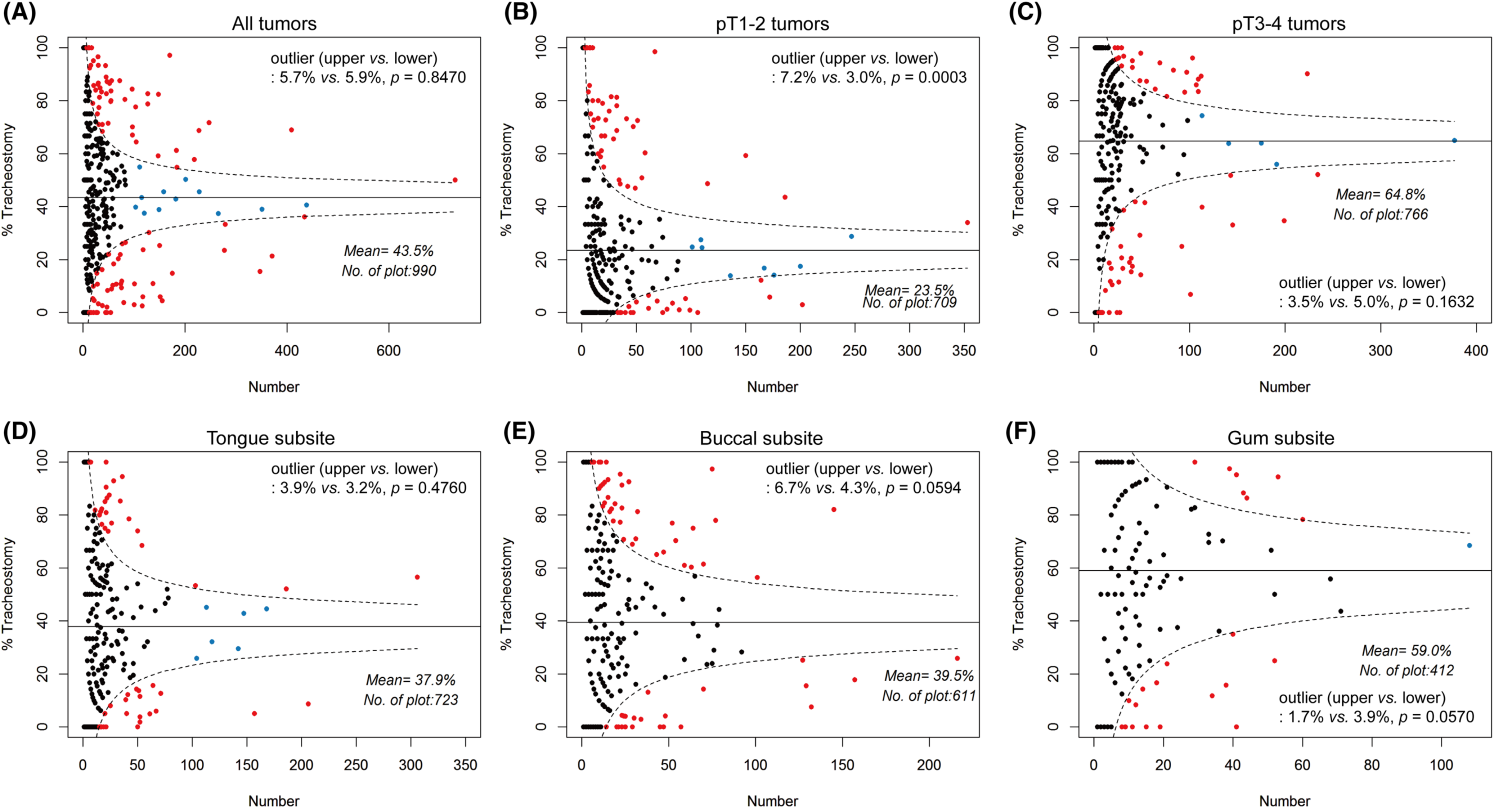
Distribution of surgeons specializing in oral cavity cancer treatment, stratified by patient number and tracheotomy rates. Panel A depicts all tumors, whereas panels B and C are focused on pT1–2 and pT3–4 tumors, respectively. The remaining panels depict different oral cavity cancer subsites, that is, tongue (panel D), buccal region (panel E), and gums (panel F), respectively.

The overall mean tracheotomy rate across registered surgeons was found to be 43.5%. Notably, 5.7% (56 out of 990) of the patients exceeded the upper outlier, whereas 5.9% (58 out of 990) surpassed the lower outlier (*p* = 0.8470; Figure 2A). When focusing on patients with pT1–2 tumors, the mean tracheotomy rate dropped to 23.5%. In this category, 7.2% (51 out of 709) of the patients exceeded the upper outlier and 3.0% (21 out of 709) surpassed the lower outlier (*p* = 0.0003; Figure 2B). In contrast, for pT3–4 tumors, the mean tracheotomy rate increased to 64.8%. Here, 3.5% (27 out of 766) of the patients exceeded the upper outlier and 5.0% (38 out of 766) surpassed the lower outlier (*p* = 0.1632; Figure 2C). In the case of the tongue subsite, the mean tracheotomy rate was 37.9%, with 3.9% (28 out of 723) and 3.2% (32 out of 723) of the patients exceeding the upper and lower outliers, respectively (*p* = 0.4760; Figure 2D). For the buccal subsite, the mean tracheotomy rate was 39.5%, with 6.7% (41 out of 611) and 4.3% (26 out of 611) of the patients surpassing the upper and lower outliers, respectively (*p* = 0.0594, Figure 2E). Lastly, in the gum subsite, the mean tracheotomy rate was 59.0%, with 1.7% (7 out of 412) and 3.9% (16 out of 412) of the patients exceeding the upper and lower outliers, respectively (*p* = 0.0570, Figure 2F).

### 3.3 Five‐year survival rates

**FIGURE 3:**
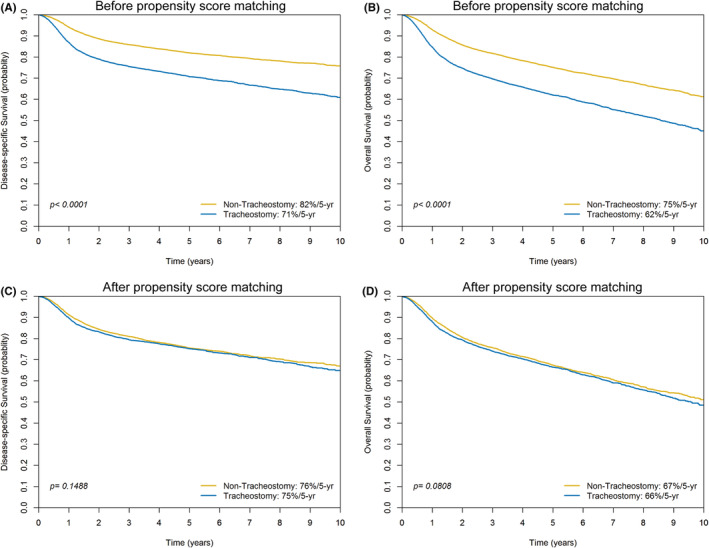
Kaplan–Meier plots illustrating the 5‐year disease‐specific and overall survival (OS) curves for patients in the original cohort (panels A and B) and after propensity score matching (panels C and D), differentiated by whether they underwent tracheotomy or not.

When comparing the 5‐year outcomes between patients who underwent tracheotomy and those who did not, significant differences were observed. The DSS rate was 71% for the tracheotomy group, significantly lower than the 82% observed in the non‐tracheotomy group (*p* < 0.0001; Figure 3A). The OS rate was also lower in the tracheotomy group at 62%, compared to 75% in the non‐tracheotomy group (*p* < 0.0001; Figure 3B).

### 3.4 Univariable and multivariable Cox regression analyses for the tracheotomy and non‐tracheotomy groups

Table 2 summarizes the results of both univariable and multivariable analyses in the original study cohort. After adjusting for potential confounders, multivariable analysis identified several factors independently associated with a decreased 5‐year DSS rate. These variables included tracheotomy (compared to non‐tracheotomy; HR = 1.10, *p* = 0.0063), tumor subsite, advanced age, pT2–4 tumors, pN1–3 disease, p‐Stage III − IV, moderately‐to‐poorly differentiated tumors, positive and close surgical margins, a deep DOI, and a weighted CCI ≥1. Similarly, several factors were independently linked to a decreased 5‐year OS rate. These included tracheotomy (compared to non‐tracheotomy; HR = 1.10, *p* = 0.0015), tumor subsite, male sex, advanced age, pT3–4 tumors, pN1–3 disease, p‐Stage III − IV, moderately‐to‐poorly differentiated tumors, positive and close surgical margins, a deep DOI, and a weighted CCI ≥1 (Table 2).

**TABLE 2:** Univariable and multivariable analyses of risk factors for 5‐year disease‐specific and overall survival (OS) in patients with and without tracheotomy before propensity score matching.

Characteristic	Disease‐specific survival				Overall survival			
	Univariable analysis		Stepwise multivariable analysis		Univariable analysis		Stepwise multivariable analysis	
	HR (95% CI)	*p*	HR (95% CI)	*p*	HR (95% CI)	*p*	HR (95% CI)	*p*
Tracheotomy								
Yes	1.81 (1.71–1.93)	<0.0001	1.10 (1.03–1.18)	0.0063	1.72 (1.63–1.81)	<0.0001	1.10 (1.04–1.17)	0.0015
No	1		1		1		1	
Tumor subsite								
Lip	1		1		1		1	
Tongue	0.93 (0.75–1.15)	0.4835	0.71 (0.58–0.88)	0.0017	0.96 (0.80–1.16)	0.6874	0.82 (0.68–0.99)	0.0357
Gum	1.20 (0.97–1.49)	0.0962	0.75 (0.60–0.93)	0.0096	1.22 (1.01–1.47)	0.0419	0.82 (0.68–0.99)	0.0423
Mouth floor	0.96 (0.74–1.24)	0.7513	0.58 (0.45–0.75)	<0.0001	1.20 (0.97–1.49)	0.0962	0.82 (0.66–1.01)	0.0652
Hard palate	1.53 (1.12–2.07)	0.0067	0.92 (0.68–1.26)	0.6058	1.73 (1.34–2.25)	<0.0001	1.20 (0.92–1.56)	0.1720
Buccal	0.90 (0.73–1.11)	0.3259	0.71 (0.58–0.88)	0.0018	0.91 (0.76–1.10)	0.3407	0.79 (0.65–0.95)	0.0103
Retromolar	1.003 (0.78–1.29)	0.9800	0.71 (0.55–0.91)	0.0067	1.02 (0.83–1.27)	0.8280	0.79 (0.63–0.98)	0.0293
Sex								
Men	1.06 (0.95–1.18)	0.2833	–	ns	1.12 (1.02–1.23)	0.0204	1.23 (1.12–1.36)	<0.0001
Women	1		–		1		1	
Age (years)	1.008 (1.008–1.011)	<0.0001	1.009 (1.006–1.011)	<0.0001	1.018 (1.015–1.020)	<0.0001	1.018 (1.016–1.021)	<0.0001
Pathologic T status								
T1	1		1		1		1	
T2	2.00 (1.77–2.27)	<0.0001	1.22 (1.002–1.49)	0.0473	1.70 (1.55–1.87)	<0.0001	1.17 (0.99–1.39)	0.0653
T3	3.17 (2.78–3.62)	<0.0001	1.31 (1.07–1.61)	0.0097	2.60 (2.34–2.89)	<0.0001	1.29 (1.09–1.54)	0.0041
T4	4.86 (4.34–5.45)	<0.0001	1.60 (1.31–1.97)	<0.0001	3.78 (3.46–4.13)	<0.0001	1.63 (1.37–1.94)	<0.0001
Pathologic N status								
pN0	1		1		1		1	
pN1	2.24 (2.12–2.58)	<0.0001	1.80 (1.60–2.03)	<0.0001	2.02 (1.86–2.20)	<0.0001	1.70 (1.53–1.88)	<0.0001
pN2	3.50 (3.21–3.81)	<0.0001	2.40 (2.15–2.67)	<0.0001	2.92 (2.71–3.14)	<0.0001	2.33 (2.12–2.56)	<0.0001
pN3	6.26 (5.82–6.73)	<0.0001	3.82 (3.47–4.20)	<0.0001	5.31 (4.99–5.66)	<0.0001	3.85 (3.54–4.19)	<0.0001
Pathologic stage								
I	1		1		1		1	
II	1.73 (1.48–2.04)	<0.0001	1.29 (1.00–1.67)	0.0503	1.51 (1.34–1.69)	<0.0001	1.18 (0.96–1.45)	0.1106
III	3.10 (2.64–3.64)	<0.0001	1.52 (1.19–1.96)	0.0010	2.44 (2.17–2.75)	<0.0001	1.37 (1.12–1.69)	0.0024
IV	6.90 (6.01–7.92)	<0.0001	1.63 (1.28–2.09)	<0.0001	4.81 (4.35–5.31)	<0.0001	1.35 (1.09–1.66)	0.0049
Tumor differentiation								
Well differentiated	1		1		1		1	
Moderately differentiated	1.67 (1.54–1.80)	<0.0001	1.24 (1.14–1.34)	<0.0001	1.57 (1.47–1.68)	<0.0001	1.24 (1.16–1.33)	<0.0001
Poorly differentiated	2.92 (2.62–3.24)	<0.0001	1.60 (1.43–1.78)	<0.0001	2.78 (2.54–3.04)	<0.0001	1.72 (1.57–1.89)	<0.0001
Margin status (mm)								
Positive	2.97 (2.68–3.29)	<0.0001	1.73 (1.55–1.92)	<0.0001	2.67 (2.44–2.93)	<0.0001	1.70 (1.54–1.86)	<0.0001
< 5	1.34 (1.25–1.43)	<0.0001	1.23 (1.16–1.32)	<0.0001	1.27 (1.20–1.34)	<0.0001	1.21 (1.15–1.28)	<0.0001
≥ 5	1		1		1		1	
Depth of invasion (mm)	1.037 (1.035–1.039)	<0.0001	1.015 (1.012–1.018)	<0.0001	1.035 (1.034–1.037)	<0.0001	1.015 (1.013–1.018)	<0.0001
Treatment modality								
Surgery alone	1		–		1		1	
Surgery plus adjuvant therapy	2.65 (2.48–2.83)	<0.0001	–	ns	2.22 (2.10–2.35)	<0.0001	0.85 (0.79–0.91)	<0.0001
Weighted Charlson comorbidity index								
0	1		1		1		1	
≥1	1.14 (1.07–1.21)	<0.0001	1.12 (1.05–1.19)	0.0006	1.32 (1.25–1.39)	<0.0001	1.25 (1.19–1.32)	<0.0001

Abbreviations: CI, confidence interval; HR, hazard ratio; ns, not significant.

### 3.5 Comparative analysis of patients with and without tracheotomy following propensity score matching

Patients who had undergone tracheotomy exhibited more severe disease compared to those who did not. To account for these differences, PS matching was applied. This resulted in two PS‐matched groups, each comprising 4301 patients (Table 1). The analysis of the PS‐matched cohort showed that the 5‐year DSS rate was similar for both groups: seventy five percentage for patients who had undergone tracheotomy and 76% for those who did not (*p* = 0.1488). Similarly, the 5‐year OS rates were 66% for patients with tracheotomy and 67% for those without (*p* = 0.0808), a difference that was marginally non‐significant (Figure 3C,D). Prior to PS matching, patients with tracheotomy had a significantly longer mean hospital stay compared to those without (23.37 ± 10.56 days vs. 14.19 ± 8.34 days; *p* < 0.0001). Following PS matching, the difference in hospital stay duration between the two groups remained significant (22.34 ± 10.25 days vs. 17.59 ± 9.54 days; *p* < 0.0001).

## 4 DISCUSSION

Elective tracheostomy is frequently performed by surgeons for patients undergoing head and neck cancer surgery to ensure optimal airway management, patient safety, and comfort during the postoperative recovery phase. However, the tracheostomy procedure itself is not typically considered to have a direct impact on the patient's cancer‐specific outcomes. Airway management is crucial in the surgical care of patients with OCSCC, as it can significantly mitigate the risk of perioperative and postoperative respiratory complications. These can stem from a variety of factors, including edema of the laryngopharynx or posterior tongue, swelling of the free flap, postoperative hemorrhage, and accumulation of secretions. To ensure a stable airway, a tracheotomy is often performed. However, tracheotomies can lead to complications such as obstruction, cannula displacement, hemorrhage, local infection, pneumonia, subglottic stenosis, and even cancerous seeding of the tracheotomy site. In 2009, Cameron et al.^13^ proposed a tracheotomy scoring system to guide airway management after head and neck surgery. This methodology takes into account factors like tumor site, mandibulectomy, neck dissection, and reconstruction. However, there is still no consensus among surgeons on the necessity of performing a tracheotomy.

In the current study, we discovered that the rate of tracheotomy varied depending on the size and subsite of the tumor. For instance, larger tumors (pT3–4) had a higher tracheotomy rate of 64.8% compared to smaller tumors (pT1–2) which had a rate of 23.5%. Similarly, the tracheotomy rate differed based on the tumor subsite, with the gum subsite having a rate of 59% compared to tongue and buccal subsites which had rates of 37.9% and 39.5% respectively. Interestingly, we observed a tendency for surgeons to perform tracheotomies more frequently than necessary in cases of smaller tumors (pT1–2) and buccal subsites, with upper outlier rates of 7.2% and 6.7%, respectively, compared to lower outlier rates of 3.0% and 4.3%. Conversely, in the gum subsite, surgeons tended to implement tracheotomies less frequently than necessary, with an upper outlier rate of 1.6% compared to a lower outlier rate of 3.9%. Therefore, the decision to perform an elective tracheotomy can vary among clinicians, as it involves a complex balance of potential benefits and risks. Some surgeons may opt for delayed extubation as an alternative, which can offer several advantages including a shorter hospital stay, reduced surgical time, and enhanced postoperative swallowing rehabilitation.^20^ Given the divergence in surgical decisions pertaining to tracheotomy, our study was designed to explore whether the patient's prognosis varied depending on the implementation of this procedure.

Our study revealed that patients who had undergone tracheotomy, compared to those who had not, exhibited a higher prevalence of risk factors. These included a higher frequency of gum, mouth floor, and retromolar tumor subsites, male sex, pT3–4 tumors, pN2–3 disease, p‐Stage IV disease, poorly differentiated tumors, positive margins, deeper DOI, treatment with surgery plus adjuvant therapy, a weighted CCI ≥1, and extended hospital stays. Prior to PS matching, the 5‐year DSS and OS rates were expectedly lower in patients who underwent tracheotomy (71% and 62%, respectively) compared to those who did not (82% and 75%, respectively; both *p* values <0.0001). However, in the original study cohort, the rate of tracheotomy increased in parallel with disease severity and poorer general patient conditions. Therefore, making a direct comparison of survival rates between patients with and without tracheotomy may result in misleading conclusions. To mitigate bias arising from confounding variables that could distort the estimate of the treatment effect, we employed PS matching. In the PS‐matched cohort, the 5‐year DSS rate was found to be similar between patients with and without tracheotomy (75% vs. 76%, respectively; *p* = 0.1488). Similarly, the 5‐year OS rate did not differ significantly between patients with and without tracheotomy (66% vs. 67%, respectively; *p* = 0.0808).

The occurrence of cancerous seeding at the tracheotomy site, while rare, can impact survival outcomes in patients undergoing surgery for OCSCC. This phenomenon is thought to occur through two primary mechanisms, that is, direct contamination or implantation of wounds with viable cancer cells, and the dissemination of circulating cancer cells due to manipulation of the primary tumor. Direct implantation can occur when surgical gloves, gowns, and instruments become contaminated with malignant cells during the resection of the primary tumor and/or neck dissection in presence of metastatic nodes. Contamination may also occur if these items come into contact with blood on the external surgical wound or are exposed to blood aspirated intra‐luminally. Due to their capacity to proliferate in active granulation tissue,^21^ the presence of malignant cells in contaminated blood within the surgical site may result in the formation of novel tumor foci.^22^ This phenomenon could explain why the tracheotomy site is a potential location for tumor recurrence, which may have implications for patient prognosis. However, in our study, PS‐matched analysis showed that the 5‐year DSS and OS rates were comparable between patients who had undergone tracheotomy and those who had not. These findings suggest that the impact of tracheotomy‐related stomal recurrence on patient survival is likely minimal or even negligible, and therefore may not be a crucial factor to consider when making treatment decisions.

Another significant clinical concern is the risk of tracheostomy‐associated pneumonia. Numerous patients with OCSCC have a history of cigarette smoking, which can compromise pulmonary function and potentially lead to chronic obstructive pulmonary disease. In such cases, the occurrence of tracheostomy‐associated pneumonia can be life‐threatening and prolong the recovery period. A study by Li et al.^23^ involving 482 oral cancer patients who underwent tracheotomy found that approximately 20% developed pneumonia. The incidence was higher in male patients and those with prolonged tracheostomy duration. This finding could explain the slightly lower OS observed in our study among patients who underwent tracheotomy compared to those who did not. To mitigate the risk of pneumonia in patients with OCSCC who had undergone tracheotomy, we recommend early removal of the tracheostomy tube when feasible.

Betel quid chewing is highly prevalent among Taiwanese patients diagnosed with OCSCC. The use of tobacco and alcohol is also common in this patient population. The reported prevalence of betel quid chewing is 20.9% in males and 1.2% in females.^24^ The significant difference in tobacco and betel quid consumption between genders may contribute to the disparate incidence rates of OCSCC observed in the Taiwanese population, with men exhibiting a substantially higher incidence (29.77/10^6^) compared to women (3.13/10^6^).^25^ The endemic practice of betel quid chewing in the Taiwanese population may also contribute to the distinct distribution of OCSCC subsites compared to other populations. In our study, 50%–55% of OCSCC cases originate from the buccal, gum, and retromolar regions,^25^ which is higher than the rates reported in Western literature. Among Taiwanese individuals with a longstanding habit of betel quid chewing, submucosal fibrosis with trismus is commonly observed in cases of buccal squamous cell carcinoma. The varying prevalence of betel quid chewing across different ethnicities and genders may account for the different incidence rates of OSCC between males and females worldwide. In Taiwan, the male‐to‐female ratio for OCSCC is 9.5:1, while in India, it is 2:1. In contrast, Western countries exhibit a more balanced ratio of approximately 1:1.^24^, ^25^ Notably, in Western countries, the oral tongue and floor of the mouth are the most common sites of OCSCC. According to the tracheostomy scoring system developed by Gupta et al.,^12^ mandibulectomy and trismus are among the risk factors for tracheostomy. Similarly, the tracheostomy scoring system proposed by Cameron et al.^13^ identifies mandibulectomy, mandibular alveolus subsite, anterior tongue subsite, and mouth floor subsite as risk factors for tracheostomy. Given the differences in the distribution of OCSCC subsites between our patient population and those in Western countries, the indications and proportions of tracheostomy for our patients are likely to differ.

Tracheotomies are recognized as a contributing factor that can extend the duration of hospitalization. This extension is attributed to both medical and surgical considerations, as well as social and logistical factors. From a clinical perspective, a newly inserted tracheal tube typically remains in place for approximately 5 days, followed by an additional period required for the safe removal of the tube. On the social and logistical front, patients who necessitate prolonged use of a tracheal tube may require intricate coordination efforts to facilitate their discharge, whether to their home or a specialized care facility. The correlation between tracheotomies and extended hospital stays is well‐documented within the medical community. Surgeons are adept at advising patients who are potential candidates for tracheotomy about the likelihood of an increased length of stay in the hospital. Despite this, the decision to proceed with a tracheostomy is often driven by compelling medical indications, making the associated extended hospitalization a justifiable and understandable outcome.

This study boasts two significant strengths. First, it encompasses the most extensive cohort to date, examining survival outcomes between patients who underwent tracheotomy and those who did not, all of whom received surgical treatment for OCSCC. This makes our findings particularly robust. Second, we utilized PS matching to mitigate bias arising from baseline intergroup differences. This methodological approach strengthens the validity of our comparison between the survival outcomes of the two patient groups. Although the study yielded negative results, it provides evidence to assure surgeons that performing an elective tracheostomy based solely on airway safety considerations does not compromise oncological outcomes. However, the interpretation of our findings warrants caution due to several limitations inherent to the study design. The retrospective nature of this cohort study precludes the establishment of a definitive causal relationship between elective tracheostomy in patients with resected OCSCC and their survival outcomes. Future prospective longitudinal studies are necessary to confirm the observed associations and further elucidate the potential causal mechanisms at play. Due to the nationwide nature of the investigation, the study datasets may contain missing data and coding errors. Another limitation is the lack of data on whether patients underwent a mandibulotomy or mandibulectomy, which are important factors to consider when deciding to perform a tracheostomy. Furthermore, the high prevalence of betel nut chewing in Taiwan, which is associated with a higher incidence of buccal subsite tumors, raises concerns about the generalizability of these findings to Western populations. We recognize that PS matching has certain limitations. These include the potential for residual confounding, the exclusion of unmatched observations from the analysis, and the possibility of violating key assumptions, which may lead to reduced statistical efficiency. Additionally, it should be noted that performing adjustments after matching can potentially introduce bias into the results. In light of these caveats, our findings should not discourage the judicious use of tracheotomy in patients who are likely to benefit from the procedure.

## 5 CONCLUSIONS

This nationwide study conducted in Taiwan revealed that, following PS matching, the 5‐year DSS and OS rates for patients who underwent tracheotomy were similar to those who did not. This suggests that an elective tracheotomy may not significantly affect survival outcome of patients with resected OCSCC, although it may associate with extended hospital stays. Therefore, when considering elective tracheotomy or prolonged intubation, surgeons should balance the potential for adverse airway events against the healthcare costs.

## AUTHOR CONTRIBUTIONS

**Ku‐Hao Fang:** Conceptualization (equal); data curation (equal); formal analysis (equal); investigation (equal); methodology (equal); project administration (equal); resources (equal); software (equal); supervision (equal); validation (equal); visualization (equal); writing – original draft (equal); writing – review and editing (equal). **Chung‐Jan Kang:** Conceptualization (equal); data curation (equal); formal analysis (equal); investigation (equal); methodology (equal); resources (equal); supervision (equal); validation (equal); visualization (equal); writing – original draft (equal). **Li‐Yu Lee:** Conceptualization (equal); data curation (equal); formal analysis (equal); investigation (equal); methodology (equal); resources (equal); supervision (equal); validation (equal); visualization (equal); writing – original draft (equal). **Shu‐Hang Ng:** Conceptualization (equal); data curation (equal); formal analysis (equal); investigation (equal); methodology (equal); resources (equal); supervision (equal); validation (equal); visualization (equal); writing – original draft (equal). **Chien‐Yu Lin:** Conceptualization (equal); data curation (equal); formal analysis (equal); investigation (equal); methodology (equal); resources (equal); supervision (equal); validation (equal); visualization (equal); writing – original draft (equal). **Wen‐Cheng Chen:** Conceptualization (equal); data curation (equal); formal analysis (equal); investigation (equal); methodology (equal); resources (equal); supervision (equal); validation (equal); visualization (equal); writing – original draft (equal). **Jin‐Ching Lin:** Conceptualization (equal); data curation (equal); formal analysis (equal); investigation (equal); methodology (equal); resources (equal); supervision (equal); validation (equal); visualization (equal); writing – original draft (equal). **Yao‐Te Tsai:** Conceptualization (equal); data curation (equal); formal analysis (equal); investigation (equal); methodology (equal); resources (equal); supervision (equal); validation (equal); visualization (equal); writing – original draft (equal). **Shu‐Ru Lee:** Conceptualization (equal); data curation (equal); formal analysis (equal); investigation (equal); methodology (equal); resources (equal); supervision (equal); validation (equal); visualization (equal); writing – original draft (equal). **Chih‐Yen Chien:** Conceptualization (equal); data curation (equal); formal analysis (equal); investigation (equal); methodology (equal); resources (equal); supervision (equal); validation (equal); visualization (equal); writing – original draft (equal). **Chun‐Hung Hua:** Conceptualization (equal); data curation (equal); formal analysis (equal); investigation (equal); methodology (equal); resources (equal); supervision (equal); validation (equal); visualization (equal); writing – original draft (equal). **Cheng Ping Wang:** Conceptualization (equal); data curation (equal); formal analysis (equal); investigation (equal); methodology (equal); resources (equal); supervision (equal); validation (equal); visualization (equal); writing – original draft (equal). **Tsung‐Ming Chen:** Conceptualization (equal); data curation (equal); formal analysis (equal); investigation (equal); methodology (equal); resources (equal); supervision (equal); validation (equal); visualization (equal); writing – original draft (equal). **Shyuang‐Der Terng:** Conceptualization (equal); data curation (equal); formal analysis (equal); investigation (equal); methodology (equal); resources (equal); supervision (equal); validation (equal); visualization (equal); writing – original draft (equal). **Chi‐Ying Tsai:** Conceptualization (equal); data curation (equal); formal analysis (equal); investigation (equal); methodology (equal); resources (equal); supervision (equal); validation (equal); visualization (equal); writing – original draft (equal). **Hung‐Ming Wang:** Conceptualization (equal); data curation (equal); formal analysis (equal); investigation (equal); methodology (equal); resources (equal); supervision (equal); validation (equal); visualization (equal); writing – original draft (equal). **Chia‐Hsun Hsieh:** Conceptualization (equal); data curation (equal); formal analysis (equal); investigation (equal); methodology (equal); resources (equal); supervision (equal); validation (equal); visualization (equal); writing – original draft (equal). **Kang‐Hsing Fan:** Conceptualization (equal); data curation (equal); formal analysis (equal); investigation (equal); methodology (equal); resources (equal); supervision (equal); validation (equal); visualization (equal); writing – original draft (equal). **Chih‐Hua Yeh:** Conceptualization (equal); data curation (equal); formal analysis (equal); investigation (equal); methodology (equal); resources (equal); supervision (equal); validation (equal); visualization (equal); writing – original draft (equal). **Chih‐Hung Lin:** Conceptualization (equal); data curation (equal); formal analysis (equal); investigation (equal); methodology (equal); resources (equal); supervision (equal); validation (equal); visualization (equal); writing – original draft (equal). **Chung‐Kan Tsao:** Conceptualization (equal); data curation (equal); formal analysis (equal); investigation (equal); methodology (equal); resources (equal); supervision (equal); validation (equal); visualization (equal); writing – original draft (equal). **Nai‐Ming Cheng:** Conceptualization (equal); data curation (equal); formal analysis (equal); investigation (equal); methodology (equal); resources (equal); supervision (equal); validation (equal); visualization (equal); writing – original draft (equal). **Tuan‐Jen Fang:** Conceptualization (equal); data curation (equal); formal analysis (equal); investigation (equal); methodology (equal); resources (equal); supervision (equal); validation (equal); visualization (equal); writing – original draft (equal). **Shiang‐Fu Huang:** Conceptualization (equal); data curation (equal); formal analysis (equal); investigation (equal); methodology (equal); resources (equal); supervision (equal); validation (equal); visualization (equal); writing – original draft (equal). **Li‐Ang Lee:** Conceptualization (equal); data curation (equal); formal analysis (equal); investigation (equal); methodology (equal); resources (equal); supervision (equal); validation (equal); visualization (equal); writing – original draft (equal). **Yu‐Chien Wang:** Conceptualization (equal); data curation (equal); formal analysis (equal); investigation (equal); methodology (equal); resources (equal); supervision (equal); validation (equal); visualization (equal); writing – original draft (equal). **Wan‐Ni Lin:** Conceptualization (equal); data curation (equal); formal analysis (equal); investigation (equal); methodology (equal); resources (equal); supervision (equal); validation (equal); visualization (equal); writing – original draft (equal). **Li‐Jen Hsin:** Conceptualization (equal); data curation (equal); formal analysis (equal); investigation (equal); methodology (equal); resources (equal); supervision (equal); validation (equal); visualization (equal); writing – original draft (equal). **Tzu‐Chen Yen:** Conceptualization (equal); data curation (equal); formal analysis (equal); investigation (equal); methodology (equal); resources (equal); supervision (equal); validation (equal); visualization (equal); writing – original draft (equal). **Yu‐Wen Wen:** Conceptualization (equal); data curation (equal); formal analysis (equal); funding acquisition (equal); investigation (equal); methodology (equal); project administration (equal); resources (equal); software (equal); supervision (equal); validation (equal); visualization (equal); writing – review and editing (equal). **Chun‐Ta Liao:** Conceptualization (equal); data curation (equal); formal analysis (equal); investigation (equal); methodology (equal); project administration (equal); resources (equal); software (equal); supervision (equal); validation (equal); visualization (equal); writing – original draft (equal); writing – review and editing (equal).

## FUNDING INFORMATION

This research received financial support through grants (CMRPD1H0521 and BMRPC55) provided by the Chang Gung Medical Research Program.

## CONFLICT OF INTEREST STATEMENT

The authors declare that there are no conflicts of interest that could potentially influence the presentation or interpretation of the research findings in this study.

## Data Availability

The availability of data used in this study is subject to third‐party restrictions imposed by the Health and Welfare Data Center of the Taiwanese Ministry of Health and Welfare (http://dep.mohw.gov.tw/DOS/), in accordance with the “Personal Information Protection Act.” Despite these limitations, the authors were granted a license to utilize the data for the purposes of this research. Access to the datasets generated and analyzed during this study can be provided by the corresponding author upon reasonable request. However, this is contingent on obtaining formal permission from the Taiwanese Ministry of Health and Welfare.
